# Application of PVA Membrane Doped with TiO_2_ and ZrO_2_ for Higher Efficiency of Alkaline Electrolysis Process

**DOI:** 10.3390/nano16010027

**Published:** 2025-12-24

**Authors:** Maslovara Sladjana, Katarina Dimic Misic, Dubravka Milovanovic, Danilo Lj Vujosevic, Andrijana Minic, Vladimir Nikolic, Milica Marceta Kaninski

**Affiliations:** 1 Institute of General and Physical Chemistry, 11158 Belgrade, Serbia; katarina.dimic.misic@aalto.fi (K.D.M.); dmilovanovic@iofh.bg.ac.rs (D.M.); daks011@yahoo.com (D.L.V.); andrijana.minic21@imperial.ac.uk (A.M.); vnikolic@iofh.bg.ac.rs (V.N.); milica@iofh.bg.ac.rs (M.M.K.); 2 Department of Chemical and Metallurgical Engineering, Aalto University, 02150 Espoo, Finland; 3 Department of Chemical Engineering, Imperial College London, London SW7 2AZ, UK

**Keywords:** PVA membrane, composites for membrane, alkaline electrolysis

## Abstract

Alkaline water electrolysis is a widely researched method for hydrogen generation due to its low cost, scalability and its advantage of being able to produce hydrogen using only renewable energy. Enhancing the efficiency of electrolysis systems relies mainly on the development of high-performance ion-conductive membranes. The incorporation of ceramic fillers into polyvinyl alcohol (PVA) membranes as a composite material has shown considerable promise in enhancing the performance of electrolyzers. In this work, novel composite separator membranes for use in alkaline electrolyzers were developed from aqueous PVA solutions and physically crosslinked through a freeze–thawing process. To enhance the membrane properties, two types of ceramic fillers—titanium dioxide (TiO_2_) and zirconium dioxide (ZrO_2_)—were incorporated into the starting crosslinking solution. The thermal stability of these membranes was studied by a Differential Scanning Calorimetry (DSC) technique where we can conclude that addition of TiO_2_ and ZrO_2_ significantly influences the thermal properties of PVA membranes. These metal oxides enhance thermal stability, as shown by the shift in exothermic peaks toward higher temperatures and alterations in the degradation mechanism, evidenced by changes in the intensity and number of DSC peaks. The effect is concentration-dependent for TiO_2_, where higher contents produce more pronounced yet increasingly complex thermal behavior. Compared with commercial membrane (Zirfon Perl), these types of membranes exhibit better electrochemical performance at ambient temperature and pressure; however, the process of preparation is simpler, reducing the cost of the hydrogen production process. The polarization curves (U-I curves) indicated a decrease in voltage with the addition of an ionic activator based on cobalt and molybdenum. Conductivity measurements performed using electrochemical impedance spectroscopy utilizing a two-probe method revealed that PVA membranes with TiO_2_ exhibit ionic conductivity comparable to that of the commercial membrane. Compared to the commercial membrane, these types of membranes demonstrated similar mechanical properties and improved electrochemical performance at ambient temperature and pressure, along with a simplified production process and lower cost of hydrogen production.

## 1. Introduction

Hydrogen production by electrolysis in alkaline electrolyzers is a mature and widely used technology, as it offers numerous advantages, including a relatively low cost of electrode materials and high process efficiency. Since the cost of the electrolytic process is very high, a lot of effort has been made to reduce energy requirements by developing electrode materials, and separator membranes and enhancing the design of the electrolytic cell [1,2,3,4,5,6]. An additional approach to reducing the cost of hydrogen production involves the use of in situ added ionic activators based on transition metals [7,8,9,10,11]. The most used diaphragm materials are asbestos-based, which are unacceptable nowadays due to their carcinogenic properties. Many other polymer membranes have been investigated, but their synthesis is based on potent carcinogenic monomers [12,13,14,15], or it has been found that such membranes do not exhibit good stability in alkaline media [16,17,18,19]. The membranes based on the Polyvinyl alcohol (PVA) polymer combine several advantages such as a simple preparation process, good stability in alkaline medium and biodegradability. PVA is a polyhydroxy polymer, which is very common in practical applications because of its easy preparation and biodegradability [10]. It has been selected as a polymer matrix in view of its film-forming capacities, hydrophilic properties and high density of reactive chemical functions favorable for crosslinking by irradiation, chemical or thermal treatments. PVA, a semicrystalline polymer with both crystalline and amorphous regions, contains hydroxyl (OH) groups that affect its physical properties, including reduced mechanical stability in water. Physical crosslinking provides a simple and cost-effective approach to enhance the strength and stability of its polymer matrix. The introduction of various ceramic fillers into polymer matrix can further enhance the mechanical properties of PVA materials [20,21,22,23]. Moreover, the addition of inorganic fillers contributes to better ionic conductivity of composite PVA material [24,25,26,27]. In our previous study, we investigated the influence of in situ added ionic activator based on d-metals on the efficiency of the electrolytic process using a commercially available Zirfon Perl membrane [10,11].

The freeze–thawing process is a physical crosslinking technique commonly used to prepare hydrogels and polymer membranes, such as PVA membranes. It involves repeated cycles of freezing and thawing an aqueous polymer solution, which induces physical crosslinking without the need for chemical crosslinking agents. It is reported that PVA gels prepared by the freeze–thaw technique resulted in higher mechanical strength in comparison with crosslinking of PVA by chemical or irradiative methods.

This study explores the feasibility of using physically crosslinked PVA membranes, enhanced with TiO_2_ and ZrO_2_ particles, as a separator membrane in alkaline electrolyzers operating with a strong alkaline electrolyte and in situ added ionic activators. TiO_2_ and ZrO_2_ are considered the most important inorganic nanoparticles due to their unique properties, such as semiconductivity with a wide energy bandgap, high refractive index, good stability, and for these reasons are utilized in various technological applications, including transparent conducting electrodes and photocatalysis [28,29]. In this research, the PVA membranes were prepared using a simple and cost-effective freeze–thawing method, which produced membranes with good mechanical properties without the need for chemical crosslinking agents, while also allowing KOH to be embedded within the polymer matrix. The performance of the alkaline electrolyzer was then evaluated using PVA/TiO_2_ and PVA/ZrO_2_ membranes in combination with an in situ added ionic activator based on cobalt and molybdenum.

## 2. Experimental Section

### 2.1. Materials and Methods

Membranes based on poly (vinyl alcohol) were prepared with two different polymer concentrations of PVA, 5 wt.% and 10 wt.%, respectively (Mw ~195,000, Aldrich, Hamburg, Germany). ZrO_2_ and TiO_2_ fillers were obtained as a dry powder from Sigma Aldrich with mass fraction of 0.5. The membranes were prepared by incorporating varying amounts of TiO_2_ and ZrO_2_ ceramic fillers into the PVA solution with the addition of either 0.5 wt.% or 1 wt.% of TiO_2_ and ZrO_2_ into the PVA matrix (Table 1).

A 5% PVA solution was prepared by dissolving 5 g of PVA in 95 mL of deionized water, named in Table 1. PVA-5 and a 10% PVA solution were made by dissolving 10 g of PVA in 90 mL of deionized water, named PVA-10 in the text. Both solutions were continuously stirred at 80 °C until fully dissolved. Afterward, 0.5 g of TiO_2_ was added to each solution and mixed constantly at 40 °C for 6 h. Ceramic particles were poured into the suspension and mixed vigorously using a high-speed Diaf mixer for 30 min (Pilvad Diaf AS, Præstemosevej 2, 4, 3480 Fredensborg, Denmark).

**Figure 1:**
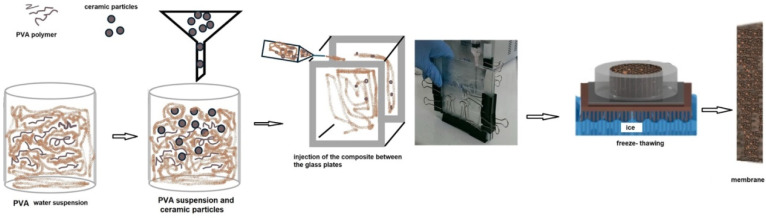
Membrane-making process.

The resulting mixtures were then poured into glass molds fitted with 2.5 mm thick gaskets. The membranes underwent four freeze–thaw cycles, each consisting of 18 h of freezing followed by 6 h of thawing. Following this, the membranes were dried in an oven at 60 °C for approximately 2 h, then further dried at room temperature for 2 days. Finally, the membranes were soaked in a 6 M KOH solution for 24 h before further use. Membranes were prepared by casting via a freeze–thawing process. Figure 1 schematically presents the process of membrane making. We use a micrometer (Micromaster series, Tesa Technology, Belgrade, Serbia) for measuring the membrane thickness at the dry state. Furthermore, the thickness of the selective PVA layers is the average value of four different locations measured on the top of the membranes.

A commercially available membrane, Zirfon UTP 500 (Agfa Geavert NV) served as a benchmark in further investigations.

**Figure 2:**
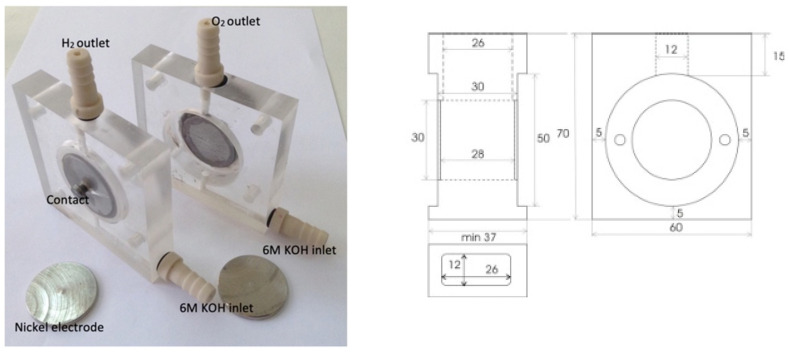
Laboratory-made alkaline electrolyzer cell.

To assess the electrochemical performance of PVA membranes doped with different ceramic fillers, TiO_2_ and ZrO_2_, a series of measurements were conducted in a house single-cell alkaline electrolyzer presented in Figure 2. For all measurements, a 6 M KOH solution (Merck) was used as electrolyte.

#### In Situ Activation of Alkaline Electrolyzer

In order to probe the performance of our composite membranes in real-life conditions, we place membrane in electrolyzer in which Co and Mo ions are added in the activation of electrodes as presented earlier research [8,10,11]. In addition to the membrane preparation, a Co-Mo-based ionic activator was added to the electrolyte solution to enhance the catalytic activity of the electrodes. The combination of two types of ionic activators were used, [Co(en)_3_]Cl_3_ and Na_2_MoO_4_ (Merck), as presented in Table 2.

Measurements were carried out using the MASTECH, DC Power Supply HY5020E, USpackage with the galvanostatic mode option. The applied current density ranged from 0.125 A·cm^−2^ to 1.25 A·cm^−2^. Before testing with the addition of ionic activators, the system with the standard electrolyte, 6 M KOH, was first tested, which then served as a reference measure for evaluating the process efficiency.

The electrolytic cell is made of plexiglass, with precisely defined geometry and appropriate fittings for the electrodes, as well as a single opening for gas. The working surface of the disk-shaped electrode is 4 cm^2^. Metal nickel with a purity of 99.9% (Zorka, Šabac, Serbia) was used as the material for both electrodes. The electrodes were treated mechanically and chemically before each measurement. The nickel electrode was highly polished with alumina paste (0.05 mm), rinsed in an ultrasonic bath for 5 min in order to remove polishing residues, degreased with acetone and rinsed with deionized water.

The construction drawing and a photograph of the cell used for the measurements are shown in Figure 2.

### 2.2. U-j Curve

The primary tool used to evaluate membrane performance was the voltage–current density (U-j) curve, which provides insight into the voltage required for a given current density. The U-j curve is a key indicator of the internal resistance of the membrane and its efficiency in transporting ions during electrolysis. The results were used to compare the PVA membranes doped with ceramic fillers to the commercial membrane in terms of voltage requirements and current-density-carrying capacity.

### 2.3. Characterization

#### 2.3.1. Raman Spectrometry

In order to evaluate the incorporation of ceramic filler in the polymer matrix, Raman spectra were collected. Raman spectroscopy was carried out using a portable version of Enspectr R532 Raman spectrometer, Austin, Texas. The Raman spectrometer is equipped with an internal laser of 532 nm excitation wavelength. The spectrometer is supplied with the objective lens, Olympus CX22 LED, Evident scientific, Tokyo, Japan, of ×10 and ×40 magnification. The output power used was 20 mW. The exposure time was set at 600 ms with 20 frames. A manual locator was used to find the spot where to place the sample on the sample holder. The measurements were performed over a range of 140–1500 cm^−1^.

#### 2.3.2. Morphology of Membranes

To evaluate the morphology of produced membranes, samples were cut to cross-section and examined with a scanning electron microscope (SEM, Hitachi TM 3000, Tokyo, Japan). Scanning electron microscopy (SEM) images were made from both pulp samples and from aerogel experimental product samples after ultrasonication in the final suspension. The preparation of the samples for SEM included the application of a thin surface layer of gold coating. Micrographs were taken using a field emission scanning electron microscope (FE-SEM, Zeiss Sigma, Carl-Zeiss-Strasse 22, 73447 Obekochen, Germany) adopting an accelerating voltage of 2.5 kV. The conventional liquid nitrogen cryogenic fracture is a commonly used method to prepare membrane cross-sections for microscopic analysis. In this method, membrane samples are cut into narrow strips and then frozen by immersion in liquid nitrogen for about few minutes. After freezing, the membranes are physically pulled apart until they break, creating fractured cross-sections.

#### 2.3.3. Differential Scanning Calorimetry (DSC)

Differential scanning calorimetry (DSC) was used to examine the thermal stability of membranes, and measurements were performed using a Setaram 151R instrument (software SETSOFT 2000 from Setaram, Caluire, France) (TA Instruments, New Catle, USA). Samples with the mass of about 3 mg were heated from 30 °C to 400 °C under nitrogen atmosphere at a heating rate of 10 °C·min^−1^.

#### 2.3.4. Electrochemical Properties

All conductivity measurements for pure PVA membranes and those doped with TiO_2_ and ZrO_2_ were performed using the electrochemical impedance spectroscopy (EIS) technique. The electrochemical properties of membranes were analyzed by EIS. The EIS measurements were carried out on a Gamry750G Potentiostat/Galvanostat/ZRE device (Palm Sens Bv, Houten, The Netherlands). The frequency range was set from 100 kHz to 0.1 Hz at voltage amplitude of 10 mV. A Teflon conductivity cell with stainless-steel electrodes was used in the experiment. The sample membrane was held between two electrodes and fixed with bolts. Before measurements, the membranes were washed in a 6 M KOH solution for about 24 h.

#### 2.3.5. Mechanical Testing

The mechanical properties such as tensile strength and elongation at break were measured on an Instron stress–strain tester, model BASF Elastollan^®^ 1185 A, Schwarzheide, Germany. After washing in a 6 M KOH solution for 24 h, samples were taken out and cut in a standard strip shape with a gauge length of 2.5 cm. A 5 N load cell was used, and the strain rate was set at 5 mm/min.

## 3. Results and Discussion

### 3.1. Raman Spectra

**Figure 3:**
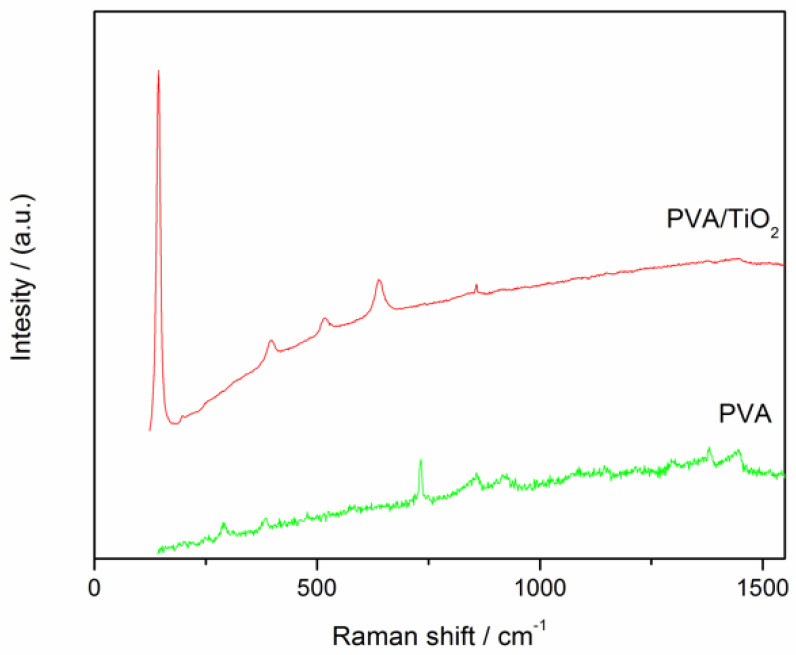
Raman spectra of PVA-10 and PVA-10-Ti membranes.

Raman spectra were collected to obtain information on the molecular composition of the prepared samples and the presence of ceramic particles in the PVA matrix. They provide more information on the phases, functions and defects. Raman spectra of reference PVA-10 membranes and PVA-10-Ti membranes are presented in Figure 3.

On both spectra, characteristic peaks of PVA are visible, at 1438 cm^−1^ (C-H and O-H bending) and 856 cm^−1^ (C-O stretching). The Raman spectrum also reveals characteristic peaks related to the anatase form of TiO_2_ at 142 cm^−1^, 396 cm^−1^, 516 cm^−1^ and 636 cm^−1^ [30], confirming the successful incorporation of TiO_2_ particles within the PVA matrix. A strong peak for the PVA polymer at 1448 cm^−1^ was due to the C–H bending and O–H bending. Moreover, the two additional vibrational peaks for the PVA polymer at 856 cm^−1^ were due to the C–C stretching. Additionally, there were several peaks at 142, 396, 516, 636 cm^−1^ for O-Ti-O and they were due to the Ti-O bonding and stretching vibrations.

The absence of standard Raman peaks for TiO_2_, ZrO_2_ and the PVA/ZrO_2_ sample may result from the low oxide content, strong interactions between the metal oxides and the PVA matrix, as well as possible overlapping or suppression of characteristic bands by the polymer background.

### 3.2. Morphology

**Figure 4:**
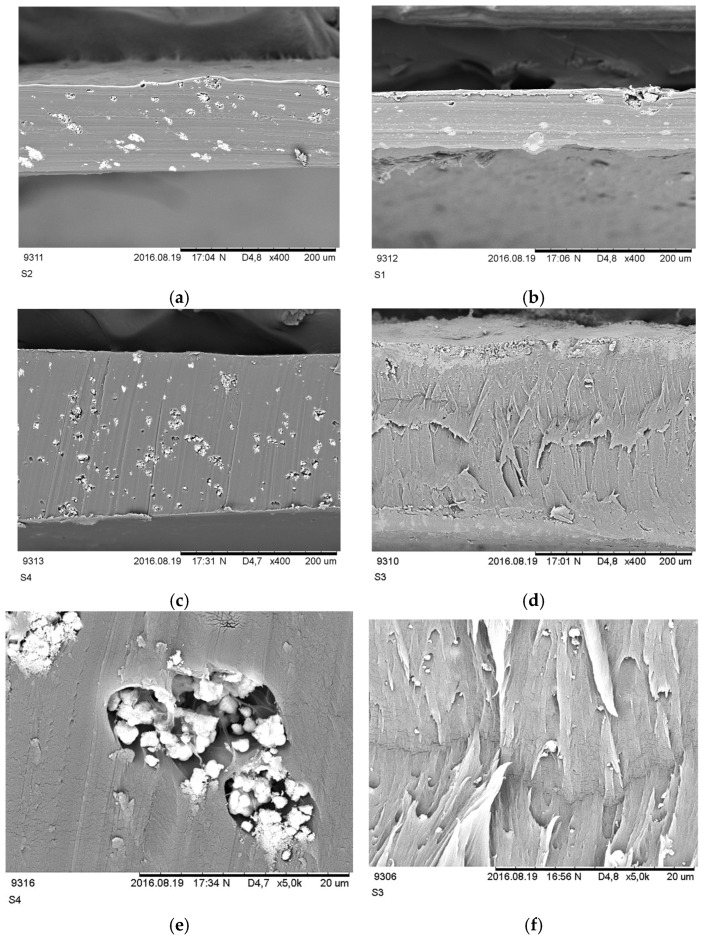
SEM pictures of cross-sections of PVA membranes: (**a**) PVA-10-Ti before soaking in KOH, magnification ×400; (**b**) PVA-10-Zr before soaking in KOH, magnification ×400; (**c**) PVA-10-Ti after soaking in KOH, magnification ×400; (**d**) PVA-10-Ti after soaking in KOH, magnification ×400; (**e**) PVA-10-Zr after soaking in KOH, magnification ×5000; (**f**) PVA-10-Zr after soaking in KOH, magnification ×5000.

SEM pictures for investigated membranes at different magnifications are shown in Figure 4, revealing the presence of ceramic particles within a uniform PVA matrix before and after soaking in 6 M KOH.

The membrane before and after soaking in 6 M KOH was imaged using an SEM device, and it was determined that no changes occurred in its structure. Ionic activators were deposited on the electrode, while the membrane maintained its ion-exchange function throughout the entire electrolytic process. SEM images of the membrane after the electrolysis process are shown in Figure 4. The membrane was imaged at a magnification of ×400 and ×5000, and a cross-section of the membrane was imaged at a magnification of ×400. The image of the membrane after soaking in 6 M KOH after the electrolytic process shows the zirconium oxide and polymer framework without disrupting the initial composition under the influence of ionic activators during the electrolytic process.

The particles of TiO_2_ ceramic filler are uniformly dispersed in the polymer matrix as shown in Figure 4c and the spherical particles of TiO_2_ are more distinguished from the rest of the PVA matrix. In contrast, ZrO_2_ particles tend to agglomerate and form spherical chunks of about 2.5 μm, which are randomly dispersed in the PVA membrane (Figure 4e).

The mechanical properties presented in Table 3 show that all prepared membranes have maximal stress before breakage of about 12 to 18 MPa, which is comparable with the results of the commercial membrane. In terms of maximum stress before breakage, better results are obtained using TiO_2_ as filler. For PVA-5-Ti and PVA-10-Ti membranes, the maximum stress is close to the value of 18.6 MPa obtained for the commercial membrane, which is one additional property that qualifies our membranes as suitable substitutes for the commercially available Zirfon Perl membrane in alkaline electrolyzers.

The mechanical properties of TiO_2_ and ZrO_2_ doped PVA membranes are enhanced due to physical crosslinking and the addition of strong filler particles into the suspension matrix.

**Table 3:** Mechanical properties of composite PVA membranes with ceramic fillers.

Membrane Samples	Maximum Load (N)	Maximum Stress (MPa)
PVA-5-Ti	17.8	18.4
PVA-10-Ti	18.8	17.4
PVA-5-Zr	10.7	14.6
PVA-10-Zr	33.8	12.4
Zirfon UTP500	79.8	18.6

### 3.3. DSC Results

**Figure 5:**
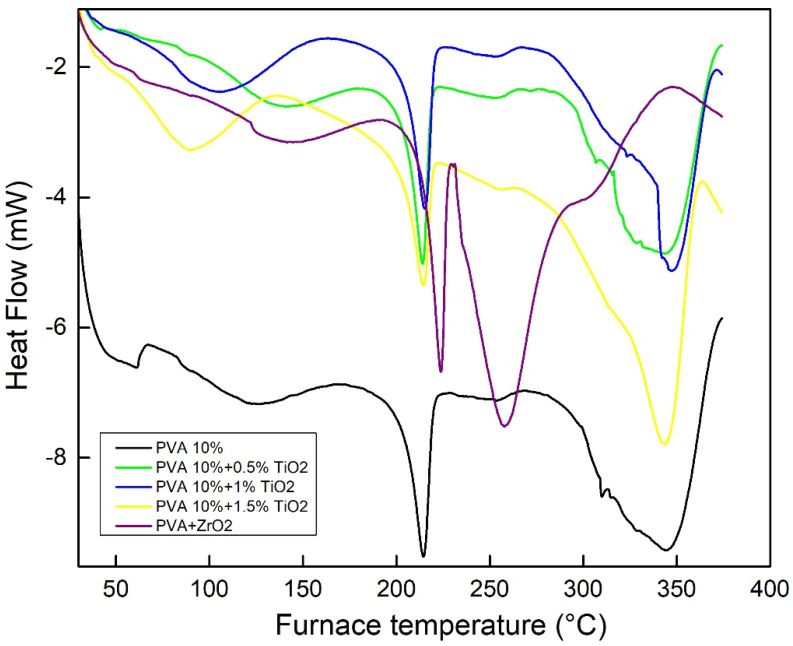
DSC curves of PVA-10, PVA-10-Ti and PVA-10-Zr membranes.

The DSC curves of PVA-10, PVA-10-Ti and PVA-10-Zr membranes are shown in Figure 5.

The glass transition (T_g_) temperature for PVA-10 and PVA-10-Ti was observed around 84 °C, while for PVA-10-Zr it was observed at 123 °C, which indicates that glass transition is increased by the addition of ZrO_2_ into the polymer matrix [31]. An endothermic peak around 215 °C (Figure 5) represents the melting temperature 1 of the examined samples (PVA-10 and PVA-10-Ti). For the PVA-10-Zr membrane, the melting temperature peak is at 223 °C, due to the change in the crystallinity. The peak that appears between 285 °C and 370 °C corresponds to the decomposition of PVA-10 and PVA10-Ti membranes. The decomposition peak of PVA-10-Zr has shifted to a lower temperature region (between 230 and 290 °C).

The temperature of the electrolytic cell greatly influences the voltage–current relationship. When operating the electrolyzer under industrial conditions—characterized by high current densities and elevated temperatures—with a basic 6 M KOH electrolyte and added ionic activators, the thermal stability of the membrane plays a crucial role.

The conductivity properties of polymer membrane samples with added TiO_2_ and ZrO_2_ are presented in Table 4.

**Table 4:** Conductivity properties for membrane samples in 6 M KOH.

Membrane Samples	Thickness (mm)	Resistance (mΩ)	Conductivity (Scm^−1^)
PVA-5	0.27 ± 0.01	590	0.014
PVA-10	0.47 ± 0.01	555	0.024
PVA-5-Ti	0.14 ± 0.01	240	0.019
PVA-10-Ti	0.36 ± 0.01	243	0.047
PVA-5-Zr	0.18 ± 0.01	373	0.015
PVA-10-Zr	0.30 ± 0.01	530	0.018
Commercial membrane	0.51 ± 0.01	274	0.059

The information about the bulk resistance (R) for different membranes is obtained from the analysis of EIS diagrams and using high frequency resistance value; conductance σ (S·cm^−1^) is calculated by the following Equation (1). Fully hydrated membranes were sandwiched in a Teflon conductivity cell equipped with electrodes having the area of 4 cm^2^. Ionic conductivity, σ (S·cm^−1^), was calculated according to Equation (1).

(1) σ = d/RA

where d is the thickness of the membrane, R is the membrane resistance and A is the geometric area of the membrane.

### 3.4. Ionic Conductivity

**Figure 6:**
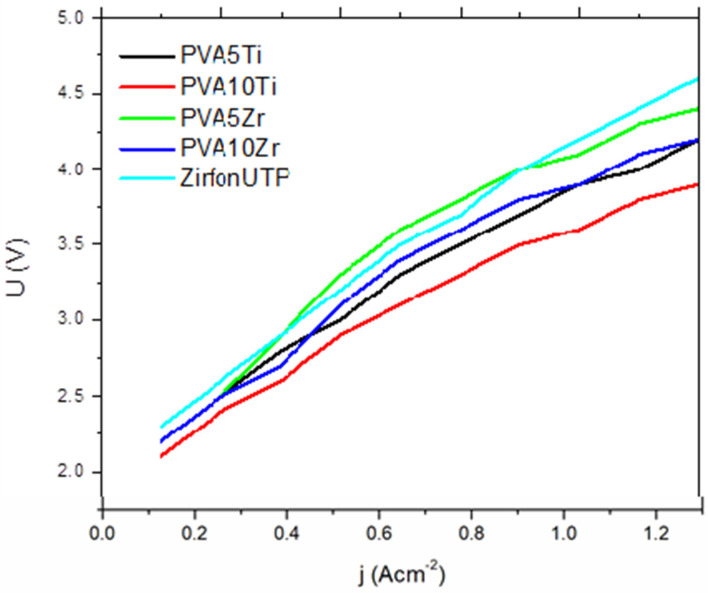
A comparison of the polarization curves in 6 M KOH for the commercial membrane and PVA membranes with ceramic fillers.

For all investigated membranes, the values of ionic conductivity were at the level of 10^−2^ Scm^−1^. Titanium dioxide (TiO_2_) ceramic fillers affect the structure of the polyvinyl alcohol (PVA) polymer by increasing the amount of the amorphous phase. This happens because TiO_2_ particles create “free volume,” or extra space, between the ceramic particles and the polymer chains. This increased free volume disrupts the tight packing of polymer chains, leading to more amorphous regions in the PVA matrix. In essence, adding TiO_2_ fillers into the PVA polymer reduces its crystallinity by creating gaps or defects at the interface between the ceramic and polymer, which allows the polymer chains to have greater mobility and less ordered arrangement, thus increasing the amorphous phase [21,32,33,34]. As a result, ionic conductivity is higher, which is also confirmed by our measurements. For example, in comparison to the pure PVA-10-Ti membrane, the addition of TiO_2_ caused the ionic conductivity to increase by a factor of two (Table 4). The recorded voltage–current densities curves, presented in Figure 6, comparatively show the performance characteristics of the investigated membranes, including those doped with TiO_2_ and ZrO_2_, as well as the commercial membrane.

In general, the results indicate that the PVA membranes doped with ceramic fillers exhibit similar voltage–current density dependence to the commercial membrane, suggesting that they are suitable for use in alkaline electrolyzers for hydrogen production. The comparison of the investigated membranes’ performance in single cell alkaline electrolyzer is achieved via a comparison of the measured cell voltage at the same applied current densities. In this way, lower voltage means lower energy requirements for the same hydrogen production rate. The U-j curves for the TiO_2_- and ZrO_2_-doped PVA membranes closely resemble those of the commercial membrane, especially at lower current densities. This suggests that, in terms of ionic conductivity and resistance to current densities flow, the ceramic-doped membranes perform similarly to the commercial membrane under typical operating conditions. However, when the current exceeds 3 A, the difference in the voltage response becomes more pronounced, with the ceramic-doped membranes exhibiting a lower voltage requirement compared to the commercial membrane. This difference may be attributed to the higher internal resistance of the ceramic fillers at higher currents, which could limit their ionic conductivity under more demanding conditions for electrolysis.

Among the ceramic-doped PVA membranes with 10 wt.%, TiO_2_, further in the text named PVA-10-Ti, showed the best performance in terms of voltage requirements across the entire range of applied current. The PVA-10-Ti membrane demonstrated consistently lower voltage values compared to the commercial membrane, indicating better overall electrochemical efficiency. This result suggests that TiO_2_, as a filler, enhances the ionic conductivity of the PVA membrane without introducing significant resistance, even at higher current densities. The superior performance of the PVA-10-Ti membrane can be attributed to the favorable interaction between the TiO_2_ particles and the PVA matrix, which is likely to enhance ion transport and lower the resistance to current flow.

While the ZrO_2_-doped PVA membranes showed some improvement over the commercial membrane, they did not perform as well as the TiO_2_-doped membranes. The ZrO_2_-doped membranes exhibited slightly higher voltage values at higher currents, which suggests that the ZrO_2_ particles might create more resistance to ion transport in comparison to TiO_2_. This difference in performance highlights the significance of careful selection of the type and concentration of ceramic fillers when optimizing membrane performance for specific electrochemical applications.

The presence of the Co-Mo ionic activator in the electrolyte solution plays a crucial role in enhancing the electrochemical performance of the system. Co-Mo is known to increase the catalytic activity of the electrode materials for the hydrogen evolution reaction (HER) [8], which, in turn, improves the overall efficiency of the electrolysis process. While the primary focus of this study was on the membrane performance, it is important to emphasize that the combination of the Co-Mo activator with the modified PVA membranes contributed to the improved hydrogen production rates observed across all membrane types.

**Figure 7:**
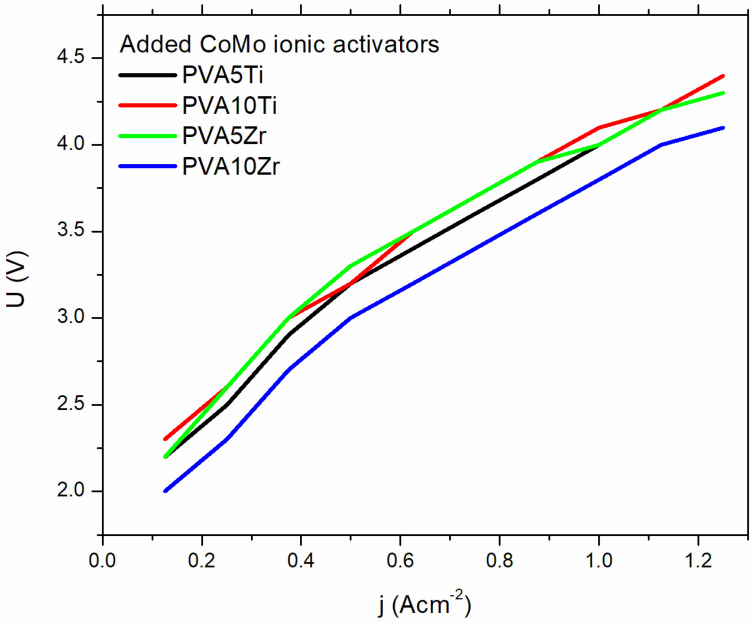
A comparison of the polarization curves with added Co-Mo ionic activator in 6 M KOH for PVA doped membranes.

To compare PVA membranes doped with different ceramic fillers, TiO_2_ and ZrO_2_, in terms of suitability for use in systems for hydrogen production with in situ added Co-Mo ionic activator into the electrolyte solution, we recorded *U-I* curves in a lab-scale alkaline electrolyzer for all membranes. All measurements were performed at room temperature and current range from 0.4 to 5 A. The voltage dependence of current for four different membranes with and without the addition of Co-Mo ionic activator into 6 M KOH is presented in Figure 7.

In situ added ionic activators decrease the value of voltage for the whole range of applied current and for all investigated composite membranes. For membranes doped with TiO_2,_ the decrease in voltage values was about 15% at lower currents. As the current increases, the difference goes down to the value of 3%. Measurements showed that the systems with ZrO_2_-doped PVA membrane are unstable, i.e., the voltage dependence of the current is not linear in the whole range of applied currents. This behavior can be attributed to the inhomogeneous distribution and agglomeration of ZrO_2_ particles within the PVA polymer matrix. The study suggests that ceramic-doped polyvinyl alcohol (PVA) membranes, particularly those incorporated with titanium dioxide (TiO_2_), present a highly promising alternative to conventional commercial membranes for applications in hydrogen production, such as in water electrolysis systems. The integration of TiO_2_ not only augments the membrane’s mechanical strength and chemical stability but also enhances its ionic conductivity and durability under alkaline conditions—key factors in achieving efficient and long-lasting performance during hydrogen generation.

Future research will prioritize optimizing the concentration of ceramic fillers to further enhance the composite membrane’s functional properties, such as its hydrogen permeability, ionic selectivity and resistance to degradation. Additionally, the exploration of various ionic activators or dopants will be undertaken to identify combinations that could further bolster conductivity or operational lifetime.

Another critical avenue of investigation will be the fabrication and evaluation of a zero-gap geometry alkaline electrolyzer utilizing these advanced membranes. The zero-gap configuration, known for minimizing internal resistance and maximizing current density, is considered pivotal for improving the overall efficiency and scalability of electrolysis systems. Successful demonstration of these membranes in such advanced setups could pave the way toward their implementation in large-scale and industrial hydrogen production, contributing significantly to the advancement of clean and sustainable energy technologies.

## 4. Conclusions

The PVA membrane with the addition of titanium (Ti) and zirconium (Zr) allows for the separation of hydrogen and oxygen gases, as well as the selective transport of hydrogen ions and water across the electrode. It enables very efficient operation of the electrolytic cell because it is stable in strong bases such as 6 M KOH. It is used as a replacement for asbestos membranes, which were previously used. This study demonstrates that the key objective of electrocatalysis—utilizing more abundant and cost-effective metals such as Co and Mo—has been largely achieved through current approaches. Additionally, the use of membranes under real-life conditions is crucial for the performance of electrolyzers when paired with these effective metals.

Under industrial alkaline electrolysis conditions, which necessitate operation at elevated current densities and temperatures, these non-noble metal electrocatalysts exhibit stable and efficient catalytic activity. The high current density environment induces significant polarization effects that accelerate reaction kinetics and hydrogen production rates, aligning with industrial requirements for scalability and economic viability [7,8,9,10,11]. Consequently, the development of electrocatalysts capable of sustaining robust performance at such industrially relevant conditions signifies a major advancement toward cost-effective and durable hydrogen production technologies.

One of the significant properties of molybdenum is its relatively high mechanical resistance at elevated temperatures, as well as a high melting point of 2610 °C. The molybdenum coating on the electrode is important in corrosion protection applications, so its influence in this combination is significant. Molybdenum cannot be directly deposited on the electrode surface from its salts in an alkaline medium. However, in the presence of metals from the iron group (Ni, Co, and Fe) and an appropriate complexing agent, co-deposition can occur.

Novel physically crosslinked PVA composite membranes were prepared by the freeze–thawing method. The influence of the addition of TiO_2_ and ZrO_2_ as inorganic fillers on overall properties of the prepared separation membranes is presented. The membranes were tested in a lab-scale alkaline electrolyzer with in situ added ionic activators based on d-metals, Co-Mo, which resulted in the reduction in the cell voltage and improvement of energy efficiency of the electrolytic process. The addition of TiO_2_ and ZrO_2_ resulted in higher ionic conductivity compared to pure PVA membranes. Satisfactory mechanical properties were obtained in comparison with a commercial Zirfon Perl membrane. From the economic point of view, PVA is a low-cost material and the preparation method of composite materials based on physically crosslinked PVA membranes with the incorporation of inorganic TiO_2_ and ZrO_2_ particles in the polymer matrix is facile and scalable.

The results of this study demonstrate that PVA membranes doped with ceramic fillers, specifically TiO_2_ and ZrO_2_, can provide performance like commercial membranes in terms of voltage-current dependence in alkaline electrolyzers. While slight differences in voltage requirements were observed at higher current densities (above 3 A), the overall electrochemical behavior of the ceramic-doped membranes suggests that they are suitable for applications in hydrogen production systems. The PVA-10-Ti membrane demonstrated the best performance across the entire range of applied current, with consistently lower voltage values compared to the commercial membrane. This indicates that TiO_2_-doping is highly effective in enhancing the ion conductivity of the PVA matrix and improving the electrochemical efficiency of the membrane.

Incorporating TiO_2_ and ZrO_2_ into PVA membranes offers several benefits for hydrogen production in alkaline electrolysis. These metal oxides enhance the thermal and mechanical stability of the membrane, reducing swelling and degradation under alkaline conditions. TiO_2_ improves ionic conductivity and provides strong interaction with the polymer matrix through hydrogen bonding, leading to better structural integrity and long-term durability. ZrO_2_ contributes to higher chemical stability and reinforces the membrane against alkaline attack, ensuring sustained performance during electrolysis. Together, TiO_2_ and ZrO_2_ improve the membrane’s conductivity, durability, and resistance to chemical and thermal stress—key factors for efficient and reliable hydrogen generation.

In conclusion, experimental results demonstrate that TiO_2_-containing PVA membranes exhibit superior properties compared to those incorporating ZrO_2_, although both materials outperform the reference PVA membrane; further higher concentrations of filler enhance membrane performance. These improvements are evident in both electrochemical and thermal properties. SEM analysis further supports these findings, revealing that the flocculation of filler particles is notably lower in the TiO_2_-based membrane than in the ZrO_2_ variant. Additionally, the enhanced particle distribution in the TiO_2_-PVA membrane contributes to a more uniform dispersion of filler particles within the PVA matrix, further optimizing its overall performance

The results demonstrate that these composite polymer membranes could be a promising alternative as separator membranes for applications in alkaline electrolyzers.

## Figures and Tables

**Table 1:** The type and composition of the membranes examined in this work.

Membrane Denotation	PVA (wt. %)	H_2_O (mL)	TiO_2_ (wt. %)	ZrO_2_ (wt. %)
PVA-5	5	95		
PVA-10	10	90		
PVA-5-Ti	5	94.5	0.5	
PVA-10-Ti	10	89	1	
PVA-5-Zr	5	94.5		0.5
PVA-10-Zr	10	89		1

**Table 2:** Ionic activators used in this research.

Component	Chemical Formula	Role	Concentration
Tris(ethylenediamine)cobalt(III) chloride	[Co(en)_3_]Cl_3_	Ionic activator	5 × 10^−3^ M
Sodium molybdate	Na_2_MoO_4_	Ionic activator	1 × 10^−2^ M

## Data Availability

The original contributions presented in this study are included in the article. Further inquiries can be directed to the corresponding author.
